# Right ventricular ejection fraction measurements using two-dimensional transthoracic echocardiography by applying an ellipsoid model

**DOI:** 10.1186/s12947-017-0096-5

**Published:** 2017-03-07

**Authors:** Stina Jorstig, Micael Waldenborg, Mats Lidén, Per Thunberg

**Affiliations:** 1 School of Medical Sciences, Faculty of Medicine and Health, Örebro University, 70182 Örebro, Sweden; 2 Department of Clinical Physiology, Faculty of Medicine and Health, Örebro University, 70182 Örebro, Sweden; 3 Department of Radiology, Faculty of Medicine and Health, Örebro University, 70182 Örebro, Sweden; 4 Department of Medical Physics, Faculty of Medicine and Health, Örebro University, 70182 Örebro, Sweden; 5 Biomedical Engineering, Örebro University Hospital, 70185 Örebro, Sweden

**Keywords:** Right ventricle, Right ventricular function, Echocardiography, Cardiac magnetic resonance imaging

## Abstract

**Background:**

There is today no established approach to estimate right ventricular ejection fraction (RVEF) using 2D transthoracic echocardiography (TTE). The aim of this study was to evaluate a new method for RVEF calculations using 2D TTE and compare the results with cardiac magnetic resonance (CMR) imaging and tricuspid annular plane systolic excursion (TAPSE).

**Methods:**

A total of 37 subjects, 25 retrospectively included patients and twelve healthy volunteers, were included to give a wide range of RVEF. The right ventricle (RV) was modeled as a part of an ellipsoid enabling calculation of the RV volume by combining three distance measurements. RVEF calculated according to the model, RVEF_TTE_, were compared with reference CMR-derived RVEF, RVEF_CMR_. Further, TAPSE was measured in the TTE images and the correlations were calculated between RVEF_TTE_, TAPSE and RVEF_CMR_.

**Results:**

The mean values were RVEF_CMR_ = 43 ± 12% (range 20–66%) and RVEF_TTE_ = 50 ± 9% (range 34–65%). There was a high correlation (*r =* 0.80, *p <* 0.001) between RVEF_TTE_ and RVEF_CMR_. Bland-Altman analysis showed a mean difference between RVEF_CMR_ and RVEF_TTE_ of 6 percentage points (ppt) with limits of agreement from −11 to 23 ppt. The mean value for TAPSE was 19 ± 5 mm and the correlation between TAPSE and RVEF_CMR_ was moderate (*r =* 0.54, *p <* 0.001). The correlation between RVEF_TTE_ and RVEF_CMR_ was significantly higher (*p <* 0.05) than the correlation between TAPSE and RVEF_CMR_.

**Conclusions:**

The ellipsoid model shows promise for RVEF calculations using 2D TTE for a wide range of RVEF, providing RVEF estimates that were significantly better correlated to RVEF obtained from CMR compared to TAPSE.

**Electronic supplementary material:**

The online version of this article (doi:10.1186/s12947-017-0096-5) contains supplementary material, which is available to authorized users.

## Background

Right ventricular (RV) function is an important predictor of survival in cardiopulmonary disease [1]. Evaluation of RV function is therefore essential in both congenital and acquired heart diseases, such as atrial septal defect, pulmonary and tricuspid regurgitation, pulmonary hypertension (PH) and arrhythmogenic right ventricular cardiomyopathy [2–4].

The complex shape of the RV, and the relatively large element of trabeculations, makes its function more challenging to assess as compared to the left ventricle (LV), when using echocardiography. Cardiac magnetic resonance (CMR) imaging is considered to be the reference method for RV evaluation allowing full ventricular coverage. In CMR, right ventricular ejection fraction (RVEF) is often used as a measure of RV function. RVEF can be determined by calculating the end-diastolic and end-systolic volumes of the RV in the short-axis (SA) plane [5]. Previous studies have shown that RVEF predicts long-term outcome in PH patients for both children and adults [6, 7].

The most common method for RV assessment is, however, transthoracic echocardiography (TTE) due to its high availability compared to CMR. A challenge when using TTE for RV evaluation is the position of the RV behind the sternum [8]. When imaging the RV in standard apical four-chamber (4CH) view shadows caused by a rib or lung often make the RV free wall hard to visualize, particularly the most apical parts. The absence of a distinct RV free wall in the TTE image makes following area and volume estimations very uncertain by both 2D and 3D TTE. For this reason tricuspid annular plane systolic excursion (TAPSE), also known as tricuspid annulus motion (TAM), is often used as a substitute for RVEF. There are, however, contradictory conclusions in the literature how well TTE-derived TAPSE correlates to CMR-derived RVEF. There are examples where the correlation between TAPSE and RVEF varies from no correlation [9, 10] to statistically significant correlation (range 0.45-0.86) [11–13]. Recent guidelines for echocardiography still recommend RV assessment by 2D TTE using multiple acoustic windows, while 3D TTE is only recommended for laboratories with experience in this area [14].

Evaluation of the RV using TTE is often made by the one-dimensional measure of TAPSE along with quantification of the RV size, often by measuring the RV diameter, and a visual evaluation of the concentric movement of the RV free wall. The fact that TTE provides a real time image of the ventricle function is an advantage, but the visual assessment might imply risk of subjectivity. Also, when using TAPSE the objective evaluation of the RV is only one-dimensional with a focus on longitudinal movement of the free wall and potential information may be left out in terms of global function. The inclusion of more than one distance measurement in the evaluation of the RVEF using TTE is a conceivable alternative for achieving a more consistent measure of RVEF which relies on a three-dimensional property (volume).

In a previous study, on healthy individuals, we have presented and evaluated a model for RV volume estimations [15], where the RV is approximated by an ellipsoid composed of three distances easily measured by TTE. The results from that study showed that the ellipsoid model underestimates RV volumes compared to reference CMR-derived RV volumes, due to underestimation of distance measurements in TTE compared to CMR. There was however a good agreement between the ellipsoid model derived RVEF and RVEF obtained from CMR. Since RVEF is calculated as a quota, it is still possible that this value can be estimated accurately even though the volumes are underestimated.

The aim of the present study was to evaluate whether the ellipsoid model can be an alternative to TAPSE for RVEF estimations by i) estimating the agreement between RVEF obtained by the ellipsoid model (RVEF_TTE_) and reference CMR-derived RVEF (RVEF_CMR_) and ii) comparing the correlation for RVEF_TTE_ and TAPSE to RVEF_CMR_ for a group of subjects with a wide range of RVEF.

## Methods

### Sample size

A power analysis was performed to calculate the sample size needed to detect differences in RVEF of 5 percentage points (ppt) between the ellipsoid model and the reference, with an estimated standard deviation (SD) of 10, power 80% and α = 0.05. The power analysis concluded a minimum of 34 subjects.

### Study population

Twenty-five patients with reduced RV function, and examined with both TTE and CMR, were retrospectively included in the study. Patients were identified in the radiology information system (RIS) using a list of all CMR examinations performed between January 2012 and May 2015. A subject was included if there was an entry of a reduced RV function in the clinical CMR report and a TTE examination had been performed within three months from the CMR examination. All subjects younger than 18 years were excluded. The criteria for inclusion in the study was met if there was a notification of reduced RV function in the CMR report defined as RVEF <50%, TAPSE <20 mm or based on visual assessment. The exact degree of reduced RV function for a specific patient was not crucial, since to the aim was to get a variety RVEF. Care was taken to ensure that the patients had not undergone any cardiac intervention of significance during the time between CMR and TTE, such as surgery and treatments including electrophysiology or potent drugs, which could have influenced on cardiac function. In this regard, care was also taken to ensure that the loading and filling conditions did not differ significantly between the two examinations such as heart rate, presence of severe valve dysfunctions and/or pericardial effusion.

In addition to the 25 patients, the twelve healthy volunteers from the previous ellipsoid study [15] were included to ensure a wide range of RVEF. These twelve examinations were consequently used in both studies. Characteristics of the subjects are presented in Table 1.

**Table 1:** Subject characteristics. Parameter values are presented as mean ± SD. Number of subjects is given as a quantity with the proportion (%) relative all subjects within brackets. Abbreviations: ARVC = arrhythmogenic right ventricular cardiomyopathy, BMI = body mass index, CMR = cardiac magnetic resonance, *n =* number, n.a. = not applicable, PH = pulmonary hypertension, RVEF = right ventricular ejection fraction, TAPSE = tricuspid annular plane systolic excursion, TTE = transthoracic echocardiography

Variables	Patients (*n =* 25)	Healthy subjects (*n =* 12)	Overall (*n =* 37)
Age (year)	55 ± 11	36 ± 12	49 ± 15
BMI [weight(kg)/length(m)^2^]	27 ± 4	24 ± 4	26 ± 4
Women	5 (20%)	4 (33%)	9 (24%)
RVEF by TTE (ellipsoid model), %	45 ± 7	59 ± 3	50 ± 9
TAPSE by TTE, mm	17 ± 5	22 ± 3	19 ± 5
RVEF by CMR (endocardial delineation in short-axis images and summation of subvolumes), %	38 ± 10	56 ± 4	43 ± 12
Time difference between CMR and TTE, days	29 ± 26	n.a.(^a^)	n.a.(^a^)
Absence of significant heart valve disease at TTE(^b^)	20 (80%)	12 (100%)	32 (86%)
Absence of significant pericardial effusion at TTE(^c^)	25 (100%)	12 (100%)	37 (100%)
Diagnosed with or suspected primarily right-sided pathology before or during the current time of study entry(^d^)	7 (28%)	0 (0%)	7 (19%)

(^a^) All healthy subjects had CMR and TTE at the same visit (separated by <30 min)

(^b^) Valve disease defined as significant stenosis and-/or regurgitation (≥ grade 2/3)

(^c^) Pericardial effusion defined as being recognized with a clear hemodynamic influence

(^d^) One patient was diagnosed with ARVC, five patients had suspected primarily right-sided pathology and one patient had biventricular dilated cardiomyopathy

The Regional Ethical Review Board approved the study and waived the informed consent requirement for the retrospectively included patients, while written informed consent was obtained from the healthy subjects.

### Examinations

The healthy subjects were examined by TTE and CMR at the same day and location, separated by less than 30 min.

All CMR examinations were performed on a clinical 1.5 T Philips Achieva system (Philips Healthcare, Best, the Netherlands). A retrospectively triggered balanced turbo field echo (b-TFE) pulse sequence was used for the acquisition of conventional images. The following parameters were applied; TR/TE shortest (typical 3.5 ms/1.7 ms), pixel size typically 1.5x1.5 mm2, flip angle 60°, 1 NSA. For the retrospectively included patients an acceleration factor (SENSE) of 2 was used, while no acceleration factor was used for the healthy subjects (SENSE = 1). For the retrospectively included patients the slice thickness/slice spacing was 5/8 mm and 30 consecutive heart phases were used for the SA images for all patients but one where 13 consecutive heart phases were used for the SA images. For the healthy subjects the slice thickness/slice spacing was 8/8 mm and 20 consecutive heart phases were used for the SA images. The protocol applied for examination of patients was slightly different from the protocol used for the healthy volunteers. The main reason for this difference is the adaptation of the protocol to a lower breath hold ability of the patients compared to healthy volunteers.

The TTE examinations of the group of patients were performed using three different types of commercial ultrasound scanners: General Electric Vivid E9 (GE Vingmed Ultrasound A/S Horten, Norway), Siemens ACUSON SC2000 (Siemens AG, Germany) or Philips iE33 (Philips Medical Systems, Andover, MA, USA). Each system was equipped with a compatible transducer (phased array and multi-frequency based). The examinations had been carried out as in clinical routine, with the subjects in the left lateral recumbent position and ECG-triggering, thus, including digital storage of moving clips (i.e. cine-loops) from standardized views, allowing offline measurements. The TTE examinations of the healthy group were performed solely using one kind of ultrasound system (GE Vivid E9). As in clinical routine, these examinations also included standardized collection procedure and digital storage of cine-loops (from standardized views).

### Ellipsoid model

The RV was approximated by an ellipsoid as previously described [15]. In short, the ellipsoid model represents the RV by an ellipsoid composed of three distance measurements available in TTE images; right ventricular inflow tract (RVIT_3_), right ventricular long axis (RVLAX) and the left ventricular maximum outer basal diameter (LVD). The right ventricular volume (RVV) is then approximated as:

$$ R V V=\frac{\pi}{6}\times R V I{T}_3\times R V LAX\times L V D $$

For a detailed derivation of the equation see Appendix. By using this estimate of the RVV, for both diastolic and systolic measurements, RVEF can be calculated.

### Measurements

**Fig. 1:**
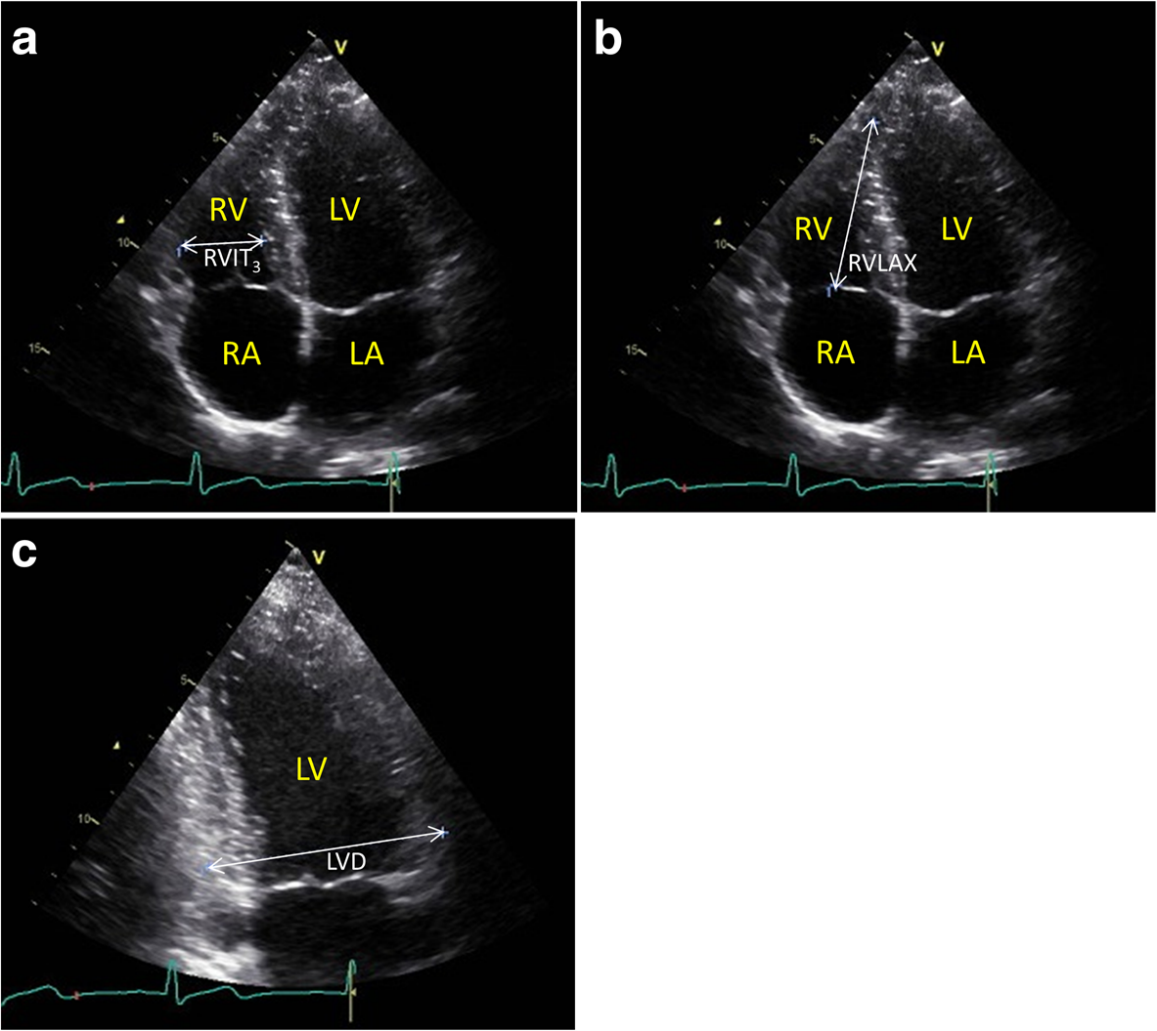
Transthoracic echocardiography distances. Images showing the transthoracic echocardiography distances for a healthy 33 year old male subject in **a**) and **b**) apical 4CH view and in **c**) apical 2CH view. LA = left atrium, LV = left ventricle, LVD = left ventricular diameter, RA = right atrium, RV = right ventricle, RVIT_3_ = right ventricular inflow tract, RVLAX = right ventricular long axis

The distances needed for the ellipsoid model, i.e. RVIT_3_, RVLAX and LVD, were measured in diastole and systole in the stored TTE images (i.e. 2D cine-loops). RVIT_3_ and RVLAX were measured in apical 4CH view, while LVD was measured in apical two-chamber (2CH) view (Fig. 1).

In addition, TAPSE was measured from M-mode TTE images as obtained in the 4CH view, for all subjects but one; where M-mode was not available. For this subject, however, TAPSE was measured in a corresponding 2D image (by so-called anatomic M-mode feature).

Offline analysis, regarding all of the TTE examinations, was done using dedicated software (EchoPAC PC, GE Vingmed Ultrasound). All TTE measurements were calculated as the average of up to three different cardiac cycles, as in clinical routine, with regard to image quality and the amount of images per view.

In SA CMR images the end-diastolic and end-systolic volumes were achieved by manually delineating the endocardium of the right ventricle using the freely available software Segment version 2.0 R4800 (http://www.segment.heiberg.se/ [16]. All available stacks with information of the tricuspid and the pulmonary valves were used to add information about the valves position in the SA images to improve the decision of which parts of the most basal slices to include in the ventricle volume. Using these end-diastolic and end-systolic volumes the RVEF was calculated.

The TTE measurements were performed by one biomedical scientist with 8 years of experience (observer 1), and one master of science in biomedical engineering with 3 years’ of experience in the scientific TTE field but limited clinical experience (observer 2). The CMR measurements were performed by one radiologist with 4 years of experience (observer3), and one master of science in biomedical engineering with 5 years’ experience in the scientific CMR field but limited clinical experience (observer 2).

Inter-observer (intra-modality) agreement for each modality was performed.

The mean value for the two observers for each method was calculated to give RVEF_TTE_ and RVEF_CMR_. These mean values were used for the inter-modality agreement evaluation.

### Statistics

Shapiro-Wilk’s test was used to determine normality of the data. Paired Student’s t-test was used to test the significance for normally distributed differences, while Wilcoxon signed-rank test was used for non-normally distributed differences. Bland-Altman limits of agreement method were used to evaluate the differences [17] for both normally and non-normally distributed differences as non-normality does not have a great impact on the limits of agreement [18]. Pearson’s correlation coefficient was used to test the correlation between the two different methods for calculating RVEF and interpreted as negligible (0 < *r <* 0.3), low (0.3 < *r <* 0.5), moderate (0.5 < *r <* 0.7), high (0.7 < *r <* 0.9) and very high (*r >* 0.9) [19]. Steiger’s z-test was used to decide whether two correlation coefficients were significantly different or not [20]. Statistical analyzes were performed using IBM SPSS Statistics version 22 (IBM Corp., Armonk, NY, USA) and Stata (version 14.1, StataCorp LP, College Station, Texas, USA). All values are presented as mean ± 1 SD. *p <* 0.05 was considered to indicate statistical significance.

## Results

The 25 retrospectively included patients had a mean age of 55 years (range 27–72 years, 20% women), while the twelve healthy subjects had a mean age of 36 years (range 18–65 years, 33% women). The TTE and CMR examinations for the 25 patients were separated by a maximum of 77 days (mean 29 days, range 0–77 days) (Table 1).

**Fig. 2:**
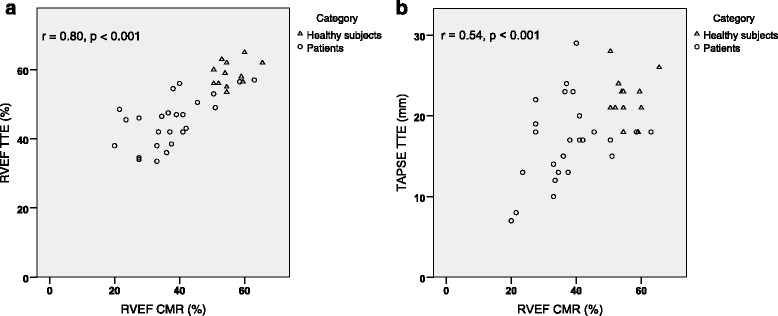
Correlation between RVEF_CMR_, RVEF_TTE_ and TAPSE. The correlation between **a**) RVEF obtained from the application of the ellipsoid model using TTE measurements and RVEF derived from CMR imaging and **b**) the correlation between TAPSE from TTE and RVEF derived from CMR. CMR = cardiac magnetic resonance, RVEF = right ventricular ejection fraction, TAPSE = tricuspid annular plane systolic excursion, TTE = transthoracic echocardiography

**Fig. 3:**
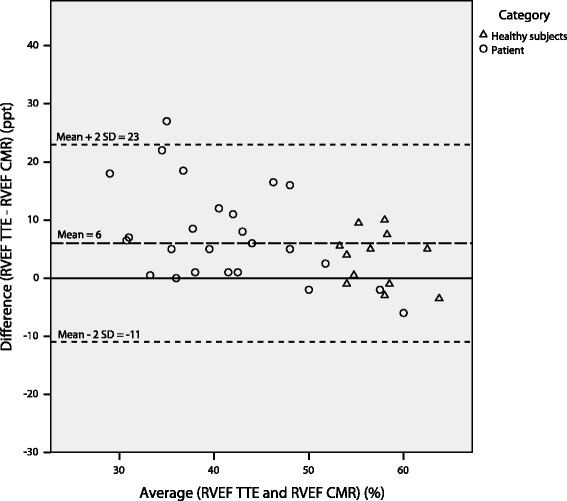
Bland-Altman plot of agreement between RVEF_TTE_ and RVEF_CMR._ Bland-Altman plot of the agreement between the mean values for RVEF calculated using the ellipsoid model on TTE measurements and RVEF derived from CMR. The dashed lines show the mean value and the limits of agreements. CMR = cardiac magnetic resonance, RVEF = right ventricular ejection fraction, TTE = transthoracic echocardiography

There was a high correlation *r =* 0.80 (*p <* 0.001) between RVEF_TTE_ and RVEF_CMR_, while the correlation between TAPSE and RVEF_CMR_ was moderate *r =* 0.54 (*p <* 0.001) (Fig. 2). The correlation between TAPSE and RVEF_CMR_ was significantly lower than the correlation between RVEF_TTE_ and RVEF_CMR_ (*p <* 0.05). RVEF_TTE_ obtained from the ellipsoid model was 50 ± 9% (range 34–65) and RVEF_CMR_ was 43 ± 12% (range 20–66). There was a significant difference between RVEF_TTE_ and RVEF_CMR_ using Wilcoxon signed rank test (Z = −4.1, *p <* 0.001). The mean value for TAPSE was 19 ± 5 mm. Figure 3 shows the Bland-Altman plot for the difference between RVEF_TTE_ and RVEF_CMR_. The standard deviation for the differences of the mean values in the Bland-Altman analysis was calculated according to the recommendation for analyzes based on mean values [17].

When the healthy subjects were excluded, the correlation between RVEF_TTE_ and RVEF_CMR_ for the remaining subgroup of 25 patients was moderate, *r =* 0.62 (*p <* 0.01), while the correlation between TAPSE and RVEF_CMR_ was low, *r =* 0.34, and could not be statistically verified. There was a significant difference (*p <* 0.001) between RVEF_TTE_ and RVEF_CMR_ for this subgroup of 8 ± 8 ppt (95% CI: 4.1-11).

The results for the RVEF calculations of each observer were RVEF_TTE obs1_ = 47 ± 10 (range 30–65), RVEF_TTE obs2_ = 52 ± 9 (range 34–70), RVEF_CMR obs2_ = 42 ± 14 (range 17–68) and RVEF_CMR obs3_ = 45 ± 12 (range 18–63). An additional file contains the RVEF values for each subject (see Additional file 1). The mean difference between RVEF_TTE obs1_ and RVEF_TTE obs2_ was 6 ± 7 ppt, while the mean difference between RVEF_CMR obs2_ and RVEF_CMR obs3_ was 2 ± 6 ppt. Both differences were normally distributed and statistically significant (*p <* 0.05). This inter-observer agreement can also be considered as an intra-modality agreement since they rely on data from the same modality. The correlation between RVEF_TTE obs1_ and RVEF_TTE obs2_ was high *r =* 0.73 (*p <* 0.001), while the correlation between RVEF_CMR obs2_ and RVEF_CMR obs3_ was very high *r =* 0.91 (*p <* 0.001).

## Discussion

Determination of RVEF is an important parameter in the assessment of cardiovascular diseases. There is today no established method for measurement of RVEF based on volume estimations using 2D TTE. The results in this study show that a combination of three conventional distance measurements in 2D TTE provides volume-based estimations of RVEF that strongly correlates to CMR for a clinically relevant range of RVEFs. The distances can easily be measured in the 2D TTE images and is not dependent on a complete visualization of the RV free wall which remains as a challenge in echocardiography. The time for measuring the distances necessary for the ellipsoid model is, after some initial testing at our department, considerably shorter compared to RVEF calculations using 3D TTE data with post processing software. The ellipsoid model is not necessarily less time-consuming compared to other conventional 2D parameters (such as TAPSE, fractional area change, longitudinal strain and strain rate by Doppler tissue imaging and speckle-tracking echocardiography (STE)), although it may be recognized with relatively high applicability in this regard; since it does not require full visualization of the entire RV free wall. The moderate correlation between TAPSE and RVEF_CMR_ was significantly lower compared to the high correlation between RVEF_CMR_ and the RVEF obtained using the ellipsoid model. This difference in correlation might seem reasonable since the ellipsoid model combines distances expanding a volume while TAPSE provides a one-directional estimate.

An upcoming method for RV evaluation is longitudinal STE strain for regional or global analysis of the RV free wall. STE strain enables quantification of the RV free wall which, compared to Doppler tissue imaging strain, is less angle dependent but the need for good image quality and full visualization of the RV free wall is still present [14]. A recent study provided reference values for RV longitudinal strain (RVLS) by STE [21]. These reference values for RVLS showed a weak correlation to RVEF by 3D TTE (0.27 for 6-segment RVLS and 0.28 for 3-segment RVLS). The authors discuss the fact that RVLS does not take into account the radial movement of the RV and that RV radial strain is difficult to measure by 2D STE [21]. Thus, at the moment this technique does not seem to add any extra value in this context (in addition to measuring TAPSE). A comparison between STE strain and the proposed ellipsoid model, regarding the assessment of the RV function, would be of interest for future studies.

There was a small but significant mean difference regarding RVEF as estimated with the ellipsoid model compared to CMR, where the ellipsoid overestimates the RVEF. As seen in Figs. 2 and 3 there are larger differences for low RVEF values compared to higher values. The limits of agreement in the Bland Altman analysis are −11 to 23 ppt. This is a rather wide range for the limits of agreement, but as shown in Fig. 3 there are six out of the 25 patients which mainly contribute to the increased mean difference and limits of agreements. Among these six patients one was diagnosed with ARVC, while four of them (of which one was recognized with severe PH) showed signs of more or less regional RV dilatation; despite not fulfilling the criteria for ARVC (Table 1). Thus, the majority of these outliers had a clearly abnormal RV morphology, indicating that the ellipsoid model is less suitable for this category of patients. However, the ellipsoid model might be used to detect RV dysfunction before it results in RV deformation. For the remaining 31 subjects however, among which there were no diagnoses of ARVC, severe PH or signs of abnormal morphology, the difference is about 10 ppt or lower, which could be considered as clinically acceptable differences.

The correlation between RVEF estimated by the ellipsoid model and CMR was lower for the subgroup of 25 patients compared to the correlation for all subjects, but still at a moderate level, while there was no correlation between TAPSE and CMR for this subgroup. This indicates that the ellipsoid model provides a better estimate of global RV function compared to TAPSE. The fact that there were up to three months between the TTE and CMR examinations for the patient group is a possible reason for the greater differences and lower correlation, along with the issue of abnormal RV morphology discussed above. Also, the number of subjects and the distribution of the RVEF-values differ when comparing the correlation coefficient for the subgroup of 25 patients to the correlation coefficient for all 37 subjects. A prospective study, performing CMR and TTE examinations on the same day for a group of subjects with a wide range of RVEF values, is needed to further evaluate the ellipsoid model.

In TTE, according to our experience, RVEF is quite often determined by visual estimation of the concentric movement of the RV free wall, along with TAPSE measurements. We believe that this new method for RVEF calculations is a way to improve the RVEF estimations using TTE, and making it less subjective.

Looking at the intra-modality agreement there was a high correlation for TTE compared to a very high for CMR. There was a slightly smaller bias and more narrow limits of agreements for CMR compared to TTE. This indication, that CMR measurements are more reproducible, agrees with CMR being considered to be the reference for such measurements. Also, the inter-observer variability may be affected by the difference in clinical experience between the observers.

CMR and TTE complement each other and a multi-modality approach is often a good alternative when possible. We believe, however, that this new method proposed for RVEF calculations using TTE may be of value when CMR is not possible.

### Limitations

One of the dimensions in the ellipsoid model is based on a left ventricular measure (LVD) and is not a direct right ventricular measure. This could influence the accuracy for groups of patients, such as patients with arrhythmogenic right ventricular cardiomyopathy (ARVC), due to the risk of an increase of the right ventricle width in this direction, which not necessarily leads to an increase of the left ventricular diameter. Further evaluation of the model’s applicability for this group of patients is necessary. An alternative to using the LVD measure in the equation, could be to replace it with the corresponding measure of the RV in a basal parasternal short-axis view, possibly resulting in better agreement also for patients with abnormal RV morphology.

When measuring RVIT_3_, the apical 4CH view should be focused on the RV, as recommended in the guidelines [14]. For the healthy subjects the RV focused apical 4CH view was used, while it is not certain that this was the case for the retrospectively included patients. This means that there is a risk of volumetric underestimation regarding the group of retrospectively included patients. However, since the RVEF is a quota, this aspect may be considered to be less significant in the context.

The fact that it was up to three months between the examinations for the retrospectively included patients might influence the results.

In this study, CMR is used as reference method. It is the method commonly used as reference method for ventricular volume calculations, but it is however important to remember that the calculations by CMR also are estimations. In particular for the RV, delineation of the endocardium is a difficult task and the true value remains unknown.

## Conclusions

The ellipsoid model provides an alternative for RVEF calculations using 2D TTE that gives a higher correlation to CMR compared to TAPSE for a wide range of RVEF. An incomplete visualization of the RV free wall, which is a common challenge in TTE, is not a restriction for the application of the method.

## Figures and Tables

**Fig. 4:**
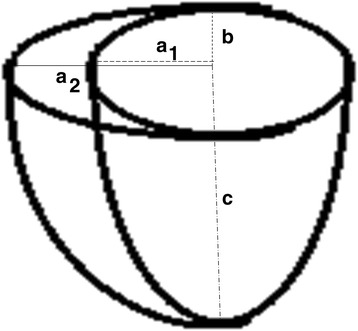
Ellipsoid model. Parts of two ellipsoids with different sizes but two of three radiuses in common

## Appendix

The area of an ellipse and volume of an ellipsoid is expressed as:

$$ A=\pi a b $$

$$ V=\frac{4\pi abc}{3} $$

where a, b and c are the radiuses of the ellipse or ellipsoid.

Figure 4 shows parts of two ellipsoids with two common radiuses, b and c. The third radius, a_1_ and a_2_ are not equal. The volume of grey region can be expressed as the difference between a quarter of the volume of the larger ellipsoid and a quarter of the volume of the smaller ellipsoid, i.e.:

$$ {V}_{grey}=\frac{\pi bc}{3}\left({a}_2-{a}_1\right) $$

If a_2_-a_1_ is approximated by RVIT_3_, b approximated by LVD/2 and c approximated by RVLAX, an estimate of RVV is given by:

$$ R V V=\frac{\pi}{6}\times R V I{T}_3\times R V LAX\times L V D $$

## Additional file

Additional file 1: RVEF calc for each observer. (XLSX 15 kb)

## Abbreviations

4CH: Four-chamber
b-TFE: Balanced turbo field echo
CMR: Cardiac magnetic resonance
LA: Left atrium
LV: Left ventricle
LVD: Left ventricular maximum outer basal diameter
PH: Pulmonary hypertension
PPT: Percentage points
RA: Right atrium
RV: Right ventricle
RVEF: Right ventricular ejection fraction
RVIT_3_: Right ventricular inflow tract
RVLAX: Right ventricular long axis
RVLS: Right ventricular longitudinal strain
RVV: Right ventricular volume
SA: Short-axis
SD: Standard deviation
STE: Speckle-tracking echocardiographic
TAM: Tricuspid annulus motion
TAPSE: Tricuspid annular plane systolic excursion
TTE: Transthoracic echocardiography
